# Trainees’ perspectives and recommendations for catalyzing the next generation of NeuroAI researchers

**DOI:** 10.1038/s41467-024-53375-2

**Published:** 2024-10-23

**Authors:** Andrea I. Luppi, Jascha Achterberg, Samuel Schmidgall, Isil Poyraz Bilgin, Peer Herholz, Maximilian Sprang, Benjamin Fockter, Andrew Siyoon Ham, Sushrut Thorat, Rojin Ziaei, Filip Milisav, Alexandra M. Proca, Hanna M. Tolle, Laura E. Suárez, Paul Scotti, Helena M. Gellersen

**Affiliations:** 1 Centre for Eudaimonia and Human Flourishing, Department of Psychiatry, University of Oxford, Oxford, UK; 2 Montreal Neurological Institute, McGill University, Montreal, QC Canada; 3 UNIQUE Neuro-AI Center, Montreal, QC Canada; 4 St John’s College, University of Cambridge, Cambridge, UK; 5 MRC Cognition and Brain Sciences Unit, University of Cambridge, Cambridge, UK; 6 Intel Labs, Santa Clara, USA; 7 Centre for Neural Circuits and Behaviou, University of Oxford, Oxford, UK; 8 Johns Hopkins University, Baltimore, MD USA; 9 Centre de recherche de l’Institut universitaire de gériatrie de Montréal, University of Montreal, Montreal, QC Canada; 10 Faculty of Biology and Institute of Quantitative & Computational Biosciences, Johannes Gutenberg University Mainz, Mainz, Germany; 11 German Research Center for Artificial Intelligence (DFKI), Kaiserslautern, Germany; 12 Independent Researcher, London, UK; 13 Harvard Medical School, Harvard University, Boston, MA USA; 14 Osnabrück University, Osnabrück, Germany; 15 University of Maryland College Park, College Park, MD USA; 16 Department of Computing, Imperial College London, London, UK; 17 Innodem Neurosciences, Montreal, QC Canada; 18 Princeton Neuroscience Institute, Princeton University, Princeton, NJ USA; 19 German Center for Neurodegenerative Diseases, Magdeburg, Germany; 20 Department of Psychology, University of Cambridge, Cambridge, UK

**Keywords:** Learning algorithms, Careers, Education

## Abstract

New developing area of NeuroAI at the intersection of neuroscience and artificial intelligence has many open challenges, one of which is training the new generation of experts. In this Comment, the authors provide resources and outline training needs and recommendations for junior researchers working across artificial intelligence and neuroscience.

## Teaser

At this critical juncture in the development of NeuroAI, we outline challenges and training needs of junior researchers working across AI and neuroscience. We also provide advice and resources to help trainees plan their NeuroAI careers.

As trainees working at the intersection of neuroscience and artificial intelligence (the nascent field of so-called NeuroAI), we are invigorated by the recent article “*Catalyzing next-generation Artificial Intelligence through NeuroAI*”^1^, wherein experts across both fields agree that “*a better understanding of neural computation will reveal fundamental ingredients of intelligence and catalyze the next revolution in AI*”. To be clear: NeuroAI is still an emerging field^2^. However, the authors of^1^ agree that to tackle the grand challenges of NeuroAI, the first priority must be to “*train a new generation of AI researchers who are equally at home in engineering/computational science and neuroscience”*^1^. The present article shares the perspectives of current NeuroAI trainees on what it may take to achieve this training objective.

**Fig. 1:**
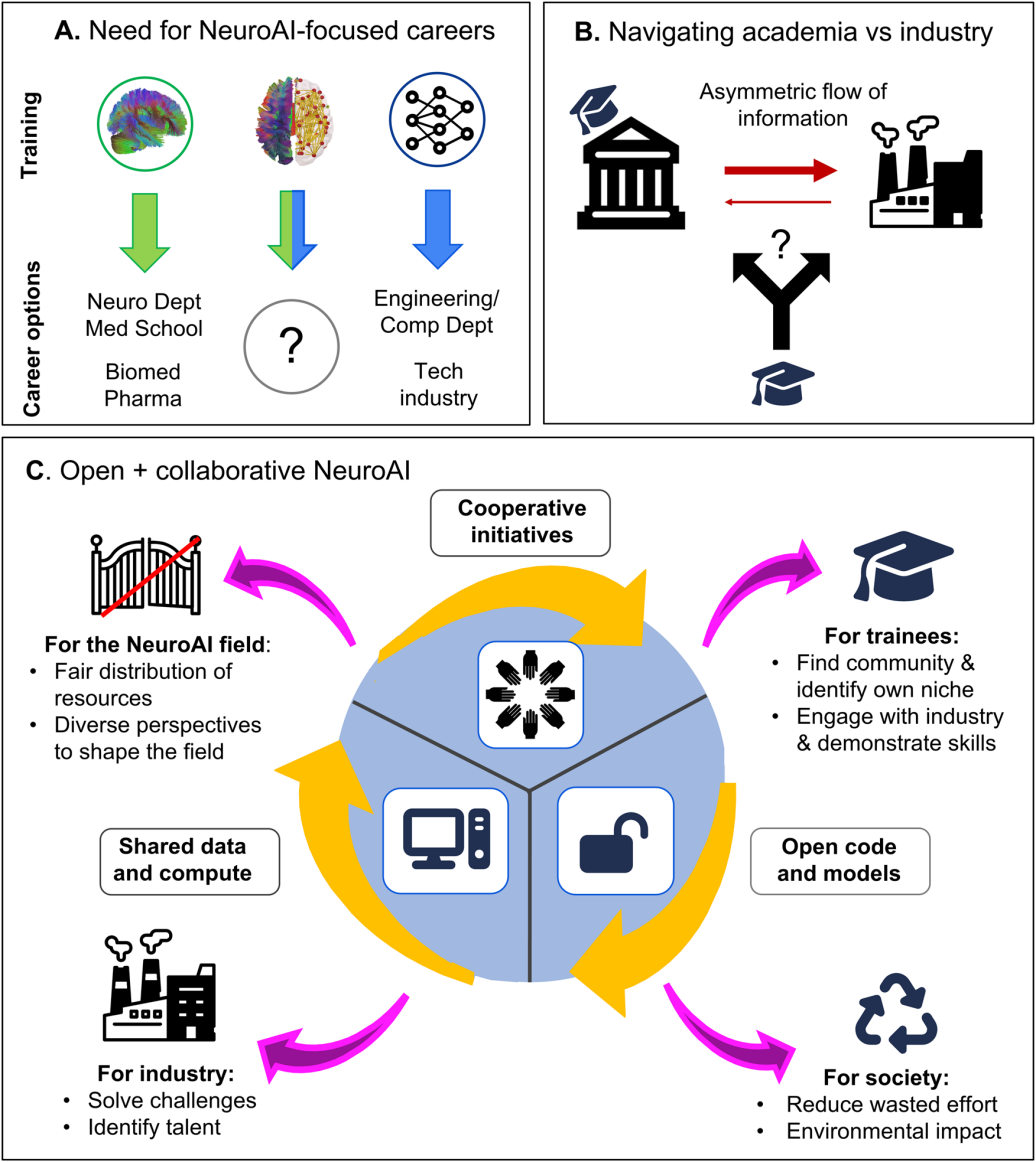
Challenges and proposed solutions for NeuroAI training. **A** There are clear-cut employment opportunities for both neuroscience trainees and AI trainees, within academia as well as in industry. However, in our experience current NeuroAI trainees are often perceived as outsiders by both sides. At the institutional level, we believe that this could be addressed by creating dedicated NeuroAI posts, rather than forcing them to fit within traditional neuroscience and computer science departments. **B** During academic training there is typically little understanding of industry’s incentives and ways of working, and the transition can feel like a leap in the dark. Trainees can take action by seeking out industry experience during their training, and by choosing mentors who explicitly support this. Institutions can take action by explicitly incorporating industry placements as part of NeuroAI training, rather than relying on external organizations or students’ resourcefulness. **C** We believe that NeuroAI as a developing field should strive to embrace open science through collaborative initiatives, open code and models, and sharing of compute and resources. This approach benefits NeuroAI trainees, who will be able to connect with the community, identify collaboration partners with complementary skills, and engage with industry. It benefits the industry by leveraging the community to address real-world challenges and identifying potential recruits. It benefits society, by reducing the need to constantly reinvent the wheel, thereby making research more efficient and reducing its environmental impact. It benefits the field of NeuroAI as a whole, by enabling greater participation from diverse perspectives, thanks to fairer allocation of resources.

**Fig. 2:**
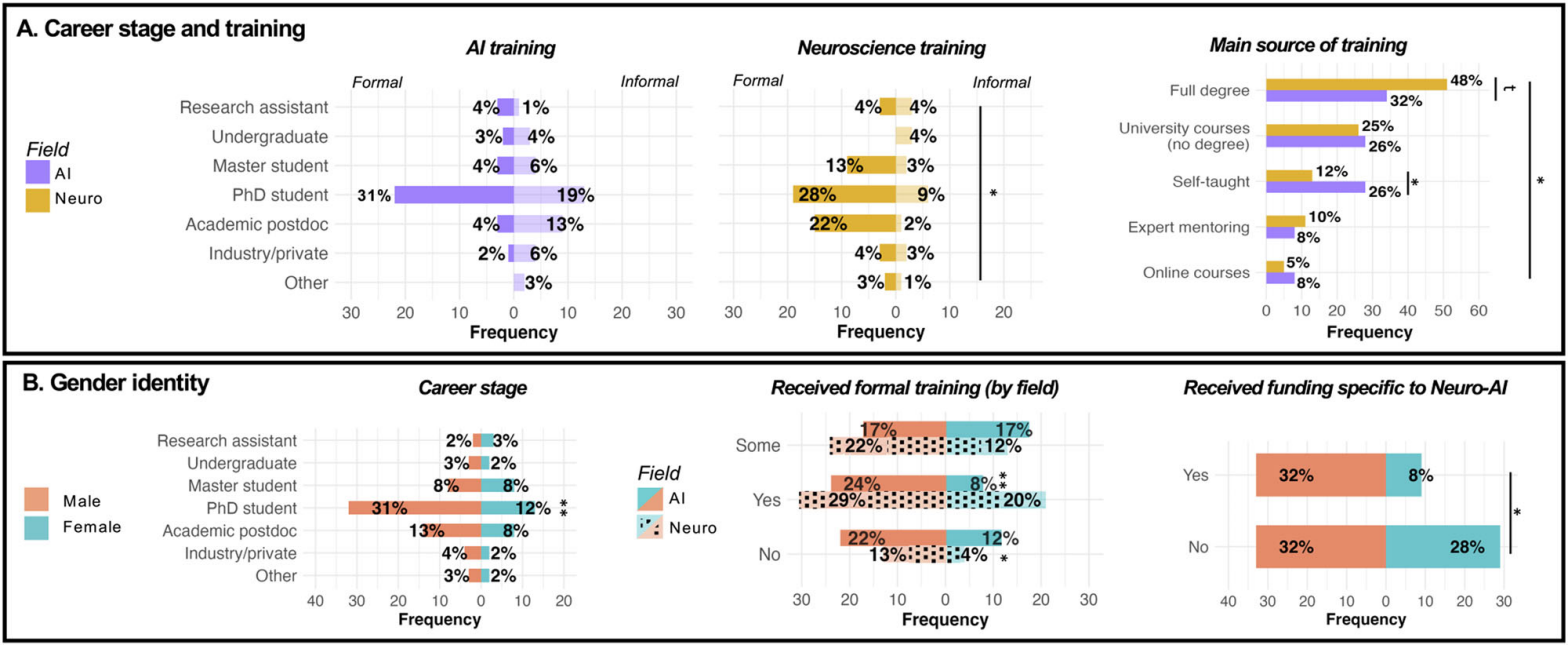
Sample of a targeted survey of NeuroAI trainees. **A** Career stage and training background. **B** Gender identity. The survey consisted of 22 questions. Full details are provided at https://github.com/8erberg/NeuroAI_Trainee_Resources. Ethical approval for the survey was sought from the Medical Sciences Interdivisional Research Ethics Committee (MS IDREC) of the University of Oxford, UK (reference number R92500/RE001). MS IDREC determined that no ethical review was required for this survey, and provided authorization to proceed. Group leaders/those holding tenure-track positions were asked to refrain from taking part, as the intention was to obtain responses from (self-identifying) NeuroAI trainees (students, postdoctoral researchers, and entry-level industry researchers). Note: Formal training = complete degree; informal training = self-taught, online courses; some formal training = university courses without a complete degree, expert mentoring. In **B,** frequencies for AI and Neuro are split by differently patterned bar graphs. Percentages within each pattern (i.e. for Neuro and AI, respectively) sum to 100%. Significance levels were obtained using χ^2^ tests where appropriate. ^t^*p* < .1, *p < .05, **p < .01, ***p < .001.

**Fig. 3:**
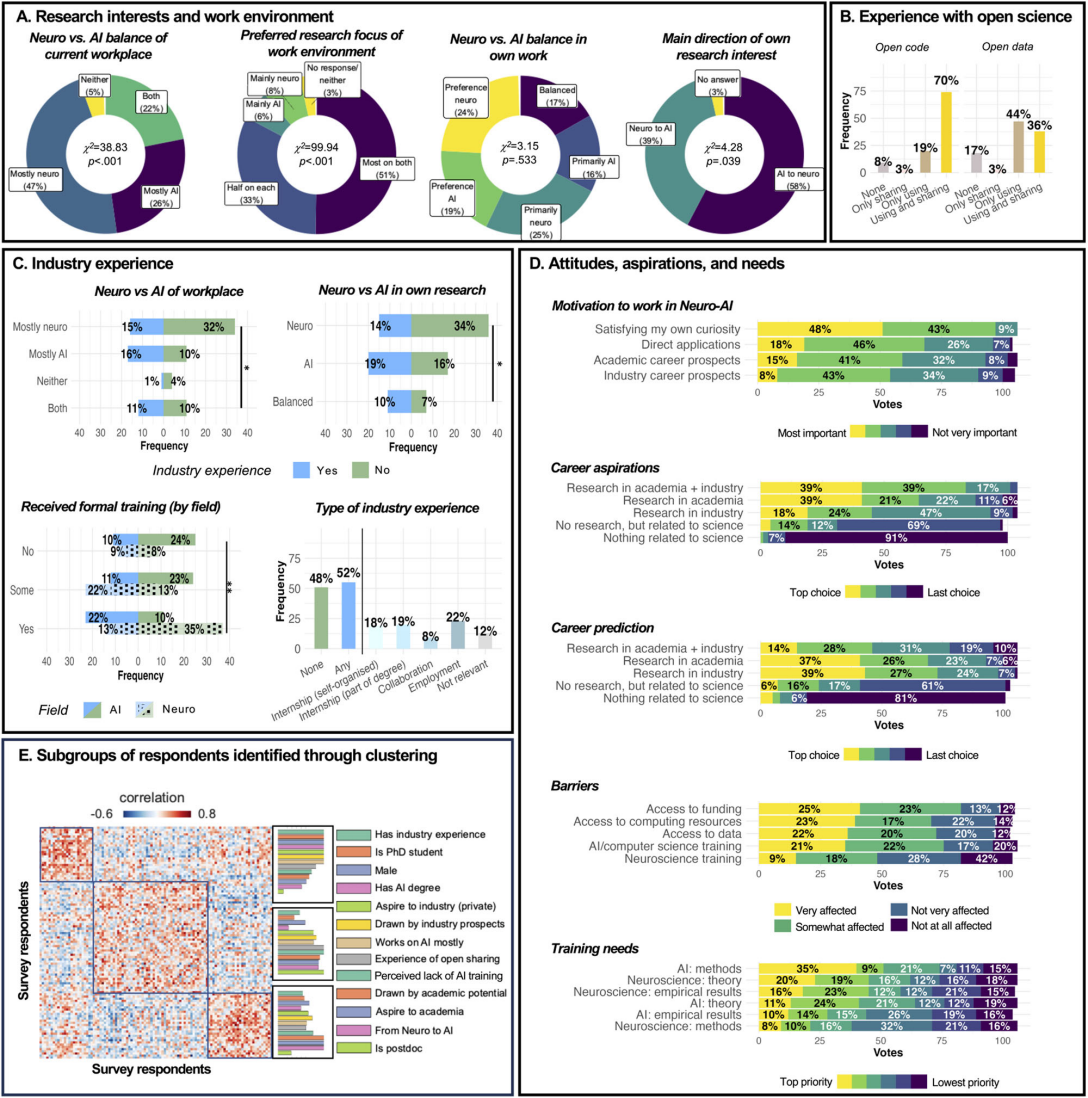
Experience and attitudes of the NeuroAI trainee sample. **A** Research interests and work environment. **B** Experience with open science. **C** Industry experience. Frequencies for AI and Neuro are split by differently patterned bar graphs. Percentages within each pattern (i.e. for Neuro and AI, respectively) sum to 100%. **D** Trainees’ attitudes, aspirations, and training needs. Labels for percentages smaller than 5% are not shown. **E** Similarity (correlation) between survey-takers’ response patterns, reordered to highlight modular structure identified by modularity-maximization, which produced 3 groups. For correlation, categorical variables were converted into one binary variable for each possible response. The response bars on the right are for visualization purposes only, intended to show which group has the maximum score on each question. See main text for exact statistics. Significance levels were obtained using χ^2^ tests where appropriate. ^t^*p* < .1, *p < .05, **p < .01, ***p < .001. Responses to individual questions in addition to those presented in this figure are shown in Figure S1 at the accompanying GitHub page for this article (https://github.com/8erberg/NeuroAI_Trainee_Resources).

Our goal is twofold: first, to provide information about NeuroAI trainees’ training needs and aspirations, at this critical juncture in the development of this fledgling field (Fig. 1). Our hope is that this information will be helpful for institutional decision-makers who are designing NeuroAI training programs, or establishing one of the growing number of NeuroAI institutes and positions. Second, we aim to help other current and prospective NeuroAI trainees by providing a “living list” of resources (https://github.com/8erberg/NeuroAI_Trainee_Resources) and our advice for planning their emerging careers in this exciting new field^3^. We also discuss a survey of NeuroAI trainees (Figs. 2 and 3), outlining the current status of available and desired NeuroAI training, background, and perceived (versus aspired) career opportunities.

## Beyond training: career prospects that recognize the synergy between AI and neuroscience

Broadly, NeuroAI wants to derive momentum from and contribute to the accelerating progress in AI, both to foster scientific understanding and technological progress. While researchers might still need to agree on a single unifying definition and methodology for NeuroAI, we here pragmatically adopt this term (following^1,2^) to refer broadly to the synergistic intersection of neuroscience and artificial intelligence, encompassing “brain-inspired AI” but also AI-oriented work in neuroscience. Even though NeuroAI is an emerging field, and NeuroAI research is broad and diverse in scope, research questions, and methodological approaches, we agree that “*the success of a NeuroAI research program depends on the formation of a community of researchers for whom the raison d'être of their training is to exploit synergies between neuroscience and AI*”^1^. The NeuroAI trainees who took part in our survey share this view: 51% wish to work in an environment where most people work on AI *and* neuroscience, while 33% prefer an environment where half of all researchers work on *either* AI or neuroscience, respectively (Fig. 3). However, instead of being “*equally at home in engineering/computational science and neuroscience”* and having access to both neuroscience and AI academic careers, in our own experience current NeuroAI trainees frequently face the challenge of being viewed as outsiders by both sides.

This is presumably a reflection of the current neuroscience-skewed status of NeuroAI training (Fig. 3): 47% of survey respondents work in neuroscience-focused institutions, with 32% having no formal AI training (i.e. online courses, self-taught) and another 34% having only some formal training (i.e. university courses, expert mentoring), compared with 17% having no formal neuroscience training whatsoever (Fig. 2). The problem of insufficient coding/programming skills in neuroscience courses has been highlighted before, with evidence that only ~15% of neuroscience PhD programs require coding, and up to one-quarter of faculty being “not at all comfortable” teaching it^4^. Indeed, 35% of survey respondents deem learning more AI methods a “top priority”, if they could, and 21% indicated being “very affected” by lack of AI credentials/training as a barrier to their ability to pursue NeuroAI work (Fig. 3).

Our survey shows that for most respondents, satisfying their own curiosity is the most (48%) or the second most (43%) important motivator for working in the field (${\chi}2$ = 45.66, *p* < .001) (Fig. 3). Similarly, a career involving academic research was rated as the top choice of 74% of trainees (${\chi}2$= 37.44, *p* < .001), with 39% of them hoping for a joint academia and industry position (Fig. 3).

Despite these agreements, a clustering approach identified 3 distinct subgroups of respondents. These three groups do not differ with respect to the Neuro vs. AI balance of their current work environment (all *p* > .14) but seem to lie along the spectrum of neuroscience to AI-based interests and applications (Fig. 3). Members of the first group (*n* = 24) of NeuroAI trainee survey-takers are primarily PhD students (18 vs. 5; χ2 = 23.55, *p* < .001) and are more likely to be male (compared to Group 2: χ2 = 8.62, *p* = .003; vs. Group 3: χ2 = 10.28, *p* = .006), have AI-related degrees (compared to Group 2: χ2 = 20.34, *p* < .001; vs. Group 3: χ2 = 20.21, *p* < .001), have funding specific to NeuroAI work (compared to Group 2: χ2 = 6.39, *p* = .011; Group 3: χ2 = 8.77, *p* = .012), have industry experience (compared to Group 2: χ2 = 5.79, *p* = .016; Group 3: χ2 = 7.33, *p* = .026), and are more likely to be working on AI compared to individuals in the third group (χ2 = 11.01, *p* = .026). The first group is also the only one in which a higher proportion of respondents are more interested in using neuroscience to build better AI systems than in applying AI methods to neuroscience problems (compared to Group 2: (χ2= 3.56, *p* = .058; compared to Group 3: χ2 = 7.27, *p* = .026). These data clearly characterize the first group as more technical, more leaning towards AI and highly industry focused (Fig. 3).

The second group is the largest (*n* = 52) and does not differ from Group 3 in terms of gender identity (χ2 = .15, *p* = .700). It is characterized by the highest proportion of academic postdocs (18 vs. four remaining postdocs across Groups 1 and 3; χ2 = 23.55, *p* < .001). Five out of the six early-career industry-based researchers were also assigned to this group, even though Group 1 had overall more industry experience with 75% of respondents having worked with or in industry compared to 48% in Group 2. Group 2 was significantly more likely to indicate that academic interests were a major motivator to work in NeuroAI (χ2 = 6.40, *p* = .011) and they were more likely to prefer a career in academia when compared to Group 1 (χ2 = 16.67, *p* < .001). However, the number of individuals who choose industry as a top career option did not differ significantly from Group 1 (*p* > .6) and their interest in direct applications of NeuroAI was significantly higher than that in the other two groups (compared to Group 1: χ2 = 4.0, *p* = .046; compared to Group 3: χ2 = 5.4, *p* = .020). They were also more likely to be concerned about not having sufficient AI training to compete with formally trained AI researchers (compared to Group 1: χ2 = 13.33, *p* < .001; compared to Group 3: χ2 = 11.65, *p* < .001) and indicated that they would most like to use additional training time for AI methods (χ2 = 20.63, *p* < .001). Compared to Group 1 they seemed to be more restricted in terms of funding despite their advanced career level (χ2 = 4.5, *p* = .034). Finally, compared to Group 3, Group 2 had more experience with open science (open data: χ2 = 10.67, *p* = .014; open code: χ2 = 6.44, *p* = .009).

Group 3 included a high proportion of students (master’s: 28%; PhD students: 45%; undergrad/assistants: 17%) and only three individuals with a completed PhD. Group 3 more strongly preferred an academic career (χ2 = 11.84, *p* < .001 compared to Group 1), and have a significantly lower proportion of individuals favouring a job in industry (1 out of 30 respondents indicated this to be their top choice; compared to Group 1: χ2 = 7.37, *p* = .006; compared to Group 2: χ2 = 5.44, *p* = .020). Even though they have a lower degree of AI training than Group 1 (χ2 = 11.31, *p* = .023), they are not concerned about insufficient training in this field (*p* > .70) and have less interest in AI methods (χ2 = 27.46, *p* < .001). However, they seem to have fewer computing resources (χ2 = 4.48, *p* = .034) and less access to data (χ2 = 4.48, *p* = .034). Their priority in terms of extra training and time are on neuroscience methods (compared to both Groups 1 and 2: χ2 = 4.50, *p* = .034), empirical results (compared to Group 1: χ2 = 6.23, *p* = .013), and most of all neuroscience theory (compared to Group 2: χ2 = 7.14, *p* = .008). Overall, this third group clearly has a strong focus on neuroscience, deems their AI training level sufficient for their academic research and is less keen on industry-based work (Fig. 3).

These data show that among survey-takers, greater neuroscience focus tends to align with greater academic interest, and greater AI focus aligns with greater interest (and experience with) industry. However, a substantial proportion of trainees in our survey occupies the middle-ground, aiming to balance both academia and industry equally. In addition to these clusters, another clear trend we show is the well-documented gender bias in STEM fields (https://www.stemwomen.com/women-in-stem-statistics-progress-and-challenges): only 8% of respondents with formal AI training were female, which could explain their greater self-reported concerns of technical AI skills and account for the significantly smaller proportion of female grant awardees for funding specific to NeuroAI (Fisher’s exact test*: OR* = 3.19 [1.23, 8.89], *p* = .012; Fig. 2). Overall, these findings of distinct subgroups show that NeuroAI is not only a diverse field in terms of ideas and methods, but also in terms of people (while acknowledging that additional dimensions of diversity also exist).

Of course, not every neuroscientist will need to have AI training, and not every AI researcher will need neuroscience training. But among trainees who wish to work at the intersection of AI and neuroscience, our data highlight the need for dedicated training on both the neuroscience and AI facets of NeuroAI. As an additional benefit, research in cognitive science suggests that interdisciplinary training can promote creativity and innovation, by providing exposure to diverse ways of thinking about similar questions, “cross-pollination” of ideas, and a broader and more flexible knowledge base^5^. We acknowledge the challenge of expanding the curriculum to span elements of both neuroscience and AI, while providing sufficient depth and rigor in both. However, we are optimistic that this can be achieved, drawing on the successful example of interdisciplinary fields such as biophysics^6^.

However, we believe that training alone will not be enough to develop NeuroAI to its full potential, if trainees are subsequently required to prioritize one identity at the expense of the other, to fit within a traditional neuroscience or computing/engineering appointment. Rather, as trainees our perspective is that NeuroAI training should be accompanied by suitable career opportunities *after* training^7^. Our hope is that recognition of the synergy between AI and neuroscience will translate into dedicated NeuroAI posts, rather than forcing NeuroAI trainees to fit within traditional neuroscience or computing departments. As a source of optimism, recent years have seen the creation of several institutes/centers (and faculty positions) with the merger of neuroscience and AI as a key focus (https://github.com/8erberg/NeuroAI_Trainee_Resources) - although dedicated NeuroAI funding for trainees seems to remain scarce to date: among the NeuroAI trainees who took our survey, 51% received no dedicated NeuroAI funding (Fig. 2), and 25% reported lack of funding to be the main barrier to their NeuroAI work (Fig. 3).

## Navigating the relationship between industry and academia

In addition to resolving the tension between the neuroscience and AI aspects of NeuroAI researchers’ identity, NeuroAI trainees will also need to navigate between academia and industry^8^ - whether in terms of choosing between them, or figuring out how to combine them^9^. Indeed, 39% of trainees in our survey rank “academia+industry” as their first-choice career, but currently only 14% expect that they will achieve their preferred career. Conversely, only 18% of respondents to our survey would have industry as their first choice, but more than twice that (39%) expect that this will be their job in 5–10 years (Fig. 3).

Therefore, in our view trainees need to “have career conversations that cover both academic and nonacademic paths”^10^. To be clear, although here we address the issue of industry experience from the perspective of NeuroAI training (see also the next section), this issue is not restricted to NeuroAI, but applies to STEM training more broadly: recent data show that “only one-third of physical and engineering sciences postdoctoral researchers and less than one-quarter of life sciences postdocs ultimately transitioned to tenure-track positions within 5–6 years”^11^, and that “most [87.6%] engineering PhD graduates will never secure a tenure-track faculty position”, including computer scientists and biological/biomedical engineers^12^. Our hope for the future of NeuroAI training is that institutions providing NeuroAI training will also prepare trainees for industry roles - not just relying on the inherent employability of the AI component, but valuing the neuroscience aspect as well.

Even for industrial R&D, the *modus operandi* of fundamental research and discovery can be at odds with commercial interests and practical applications. Making an informed decision about whether and how to engage with industry demands a resolution of the present informational asymmetry between academia and industry.

Among NeuroAI trainees who took our survey, 48% have no experience in a relevant industry (Fig. 3). This is most prominent among those working in neuroscience-focused institutions (χ^2^ = 6.69, *p* = .035) and whose own research centers more on neuroscience than AI (χ^2^ = 8.07, *p* = .018). When comparing the amount of different types of industry experience by trainee (such as internships, collaborations, and employment), only individuals without *any* formal AI training had fewer experiences than those with a full technical degree (ANOVA: *F*(2,103) = 3.40, η^2^ = .06; no AI formal training vs. AI degree: *t*(103) = −2.55, *p* = .033), while those with some formal training (university courses or expert mentoring) did not differ from individuals with a full technical degree in terms of industry experience (*t*(103) = −1.79, *p* = .177).

This lack of familiarity with industry can hinder communication and collaboration, making the prospect of a transition feel like a leap in the dark. We hope that NeuroAI training programs of the future will explicitly incorporate industry placements and business training for *all* trainees. Current trainees can already take action, however, by seeking out industry experience during their training (e.g., through Canada’s MITACS; UK Industrial Co-operative Awards in Science and Technology studentships; Royal Society’s Short Industry Fellowship Program). Prospective NeuroAI trainees should check whether their chosen mentors support trainees’ professional development through engagement with industry^10^.

Including working experience outside the academic environment as part of training would be helpful for nurturing trainees’ ability to sense and pursue promising new research directions, in terms of real-world impact and applications. Trainees would benefit from understanding companies’ incentives and how industry positions work, to develop more tailored skills, and make an informed career choice. Companies would benefit from having access to more industry-ready talent, and academia would benefit from establishing closer ties and collaborations with industry. We believe that showcasing NeuroAI trainees’ skills through industry placements should also be a goal for educational institutions that are seriously committed to student employability.

## Openness, and cooperation over competition

For the AI side specifically, the growing trend towards increasingly larger and deeper model architectures imposes an additional barrier, in terms of obtaining (i) the required large-scale training datasets; and (ii) the computational resources to run the training. Open sharing of pre-trained model weights can greatly contribute to alleviating these barriers, as well as helping best practices to spread. The resulting standardization of the field facilitates accessible training for trainees from a variety of backgrounds while diminishing the need for labs and companies to re-train talent according to idiosyncratic in-house practices.

The benefits of open science, from increased reproducibility to avoiding wasted effort, are well-documented^13^. In fact, the vast majority of survey respondents had experience with either using or sharing open code (92%) and open data (83%), which demonstrates that NeuroAI trainees recognize these benefits and share a commitment to reproducible research and resource sharing (Fig. 3). However, this open model highlights a potential tension with industry practices. In the authors’ view, resolving this tension could involve switching to a more cooperative approach to the development of AI models. Efforts to develop open-source versions of closed-source models push in this direction by undermining the value of keeping one’s models closed-source in the first place. More broadly, cooperation between research groups, and between industry and academia, can take the form of hackathons (https://brainhack.org/)^14^ or contests (e.g., Animal-AI Olympics^15^, Kaggle https://www.kaggle.com/; ML Reproducibility Challenge https://paperswithcode.com/rc2022; Algonauts Challenge http://algonauts.csail.mit.edu/), which have historically already contributed to the development of AI advances, and notably also to uniform standards and benchmarks. Industry-involving decentralized initiatives (https://github.com/8erberg/NeuroAI_Trainee_Resources) have been helping researchers to share knowledge, find synergies, and coordinate bottom-up joint projects - including the present article, which originated and coordinated on the OpenBioML platform.

Given the substantial environmental costs incurred when running such large-scale computation^16^, avoiding the need to re-train a model from scratch carries broader societal relevance. More data sharing will also allow for more diverse datasets for training AI models, helping to avoid algorithmic bias and unintentional convergence of AI systems’ cognitive profile towards specific cultures^17^—an issue that is also present in neuroscience, with much early research focusing on WEIRD (Western, Educated, Industrialized, Rich, and Democratic) groups^18^. By facilitating the integration of diverse datasets, open data sharing can contribute to more nuanced understanding of the cultural dimensions of cognition, and help to build AI systems that are more attuned to cultural diversity. Additionally, making training data, model weights, and computing resources more accessible enables broader participation in the NeuroAI endeavor, especially from less privileged/affluent backgrounds that may have limited access to large-scale computing and data-collection resources. Access to data or compute, are each rated as the number-one barrier to NeuroAI work by over 20% of survey-taking trainees: comparable with lack of funding. Open sharing of resources can alleviate this need and complement online training courses such as Neuromatch Academy^19^ (which has a NeuroAI training course as of 2024) and Neurohackademy (https://github.com/8erberg/NeuroAI_Trainee_Resources), bringing a greater diversity of perspectives to NeuroAI by lowering the existing barriers. NeuroAI being a nascent field, we believe that it will be key to avoid early lock-in of ideas by a few parties, merely due to their greater access to funding.

## Summary of NeuroAI trainees’ advice and desiderata

In this piece, we aimed to outline some of the challenges that NeuroAI trainees may currently face along their career trajectories, as reflected in our survey and the experience of our group of authors. We provided our suggestions for how trainees and educational institutions could act to address these challenges. We were motivated by the words of Ganapati and colleagues^20^: “The perspectives of current STEM Ph.D. students and recent Ph.D. alumni are important to inform the modernization of Ph.D. programs in order to facilitate job transitions, [and] provide accountability to students”. Although we do not claim to speak for all NeuroAI trainees, we hope that our experiences and opinions together with the insights obtained from our survey will be useful in shaping the development of this exciting field.

Desiderata for the future of NeuroAI training and post-training trajectory:

- Explicitly incorporate industry experience in NeuroAI programs;
- More explicit institutional cooperation between neuroscience and computing departments, for both training and hiring;
- Open up dedicated career opportunities for NeuroAI researchers;
- Facilitate open sharing of data and resources.

Advice for current and prospective NeuroAI trainees:

- If dedicated training in AI or neuroscience is unavailable, supplement with open resources, and online courses; a list is provided in our community resources on GitHub (https://github.com/8erberg/NeuroAI_Trainee_Resources). This is a “living document” that we hope will become a starting point for sharing knowledge across the NeuroAI community: anyone is encouraged to suggest new additions via pull request.
- Actively seek out industry experience, and choose mentors who are supportive of this;
- Join and contribute to open-source joint industry-academia initiatives (https://github.com/8erberg/NeuroAI_Trainee_Resources) through open platforms, as a way of engaging with peers and industry practices, and showcasing skills.

## Data Availability

We share the anonymised data from our survey of NeuroAI trainees on GitHub: https://github.com/8erberg/NeuroAI_Trainee_Resources.
